# MALDI spectrometry for salivary samples analysis : a new tool for TTR amyloidosis diagnosis

**DOI:** 10.1186/1750-1172-10-S1-P46

**Published:** 2015-11-02

**Authors:** Julie Seguier, Claude Villard, Laurie Anne Maysou, Gilbert Habib, Annie Verschueren, Pauilne Belenotti, Daniel Lafitte, Jacques Serratrice

**Affiliations:** 1 CHU Timone, Médecine Interne, 1/2, Marseille, France; 2 INSERM UMR 911, Plateforme protéomique PIT2, 2, Marseille, France; 3 CHU Timone, Service de Cardiologie, 3, Marseille, France; 4 CHU Timone, Service de neurologie, 4, Marseille, France; 5 Hopitaux Universitaires de Genève, Service de Médecine Interne Génerale, 5, Genève, Suisse

## Background

Amyloidosis suffers from a lack of accurate diagnosis tools. It results from a wrong folding of specific proteins and their identification is essential for proper medical care. Today most patients cases are identified thanks to immunohistochemistry analysis after surgery or biopsy on the defective tissues. The aim of our study is to show that diagnosis and typing of TTR amyloidosis can be immediately and rapidly achieved on formaline fixed and paraffin embedded biopsy samples using MALDI spectrometry, and also on salivary samples.

## Methods

Four fresh or formalin fixed and paraffin embedded Myocardial and salivary glands samples were analyzed. A specific de-waxing protocol using trypsic digestion and antigen retrieval was used for paraffin embedded samples. After CHCA matrix deposit, MALDI mass spectromerty acquisition was performed using MALDI-TOF, MALDI-TOF-TOF et MALDI QIT-TOF. bottom approaches using Electrospray mass spectrometry on high resolution orbitrap instruments was also performed on salivary samples.

## Results

On tissue samples the m/z ratio peak was 1366.78. Its MS/MS analysis allows to obtain m/z 1348.70, 1192.59, 1045.54, 946.46 ions, in other words, the following 4 ions coming from the 22-34 TTR-peptide GSPAINVAVHFR. On salivary samples the m/z ratio was 15991 matching with transthyretin. After MS/MS fragmentation, m/z 1047.5113 and 1051.5232 ions were identified corresponding respectively to Wild Type TTR and mutated THR 49ILE.

## Conclusions

TTR is responsible for amyloid deposits. Formalin fixed and paraffin embedded samples can be ex post analyzed after a specific de-waxing protocol by MALDI mass spectroscopy. Mutated TTR can also be identified on salivary samples. Such an approach has to be evaluated in further studies.

